# A novel sphingosylphosphorylcholine and sphingosine-1-phosphate receptor 1 antagonist, KRO-105714, for alleviating atopic dermatitis

**DOI:** 10.1186/s12950-020-00244-6

**Published:** 2020-05-29

**Authors:** Sae-Bom Yoon, Chang Hoon Lee, Hyun Young Kim, Daeyoung Jeong, Moon Kook Jeon, Sun-A Cho, Kwangmi Kim, Taeho Lee, Jung Yoon Yang, Young-Dae Gong, Heeyeong Cho

**Affiliations:** 1 Drug Discovery Platform Research Center, Therapeutics & Biotechnology Division, Korea Research Institute of Chemical Technology, 141 Gajeong-ro, Yuseong, Daejeon, 34114 Republic of Korea; 2 Department of Laboratory Animal Medicine, Research Institute for Veterinary Science, BK21PLUS Program for Creative Veterinary Science Research, College of Veterinary Medicine, Seoul National University, Seoul, 08826 Republic of Korea; 3 College of Pharmacy, Danguk University, 119 Dandae-ro, Cheonan, Chungnam, 31116 Republic of Korea; 4 Research Institute of Pharmaceutical Sciences, College of Pharmacy, Kyungpook National University, Daegu, 702-701 South Korea; 5 Innovative Drug Library Research Center, Science College, Dongguk University, Seoul, 100-715 Republic of Korea; 6 Medicinal Chemistry and Pharmacology, Korea University of Science and Technology, Daejeon, Republic of Korea

**Keywords:** Sphingosylphosphorylcholine, Sphingosine-1-phosphate receptor 1, Antagonist, Anti-inflammatory, Atopic dermatitis, High-throughput screening

## Abstract

**Background:**

Atopic dermatitis (eczema) is a type of inflammation of the skin, which presents with itchy, red, swollen, and cracked skin. The high global incidence of atopic dermatitis makes it one of the major skin diseases threatening public health. Sphingosylphosphorylcholine (SPC) and sphingosine-1-phosphate (S1P) act as pro-inflammatory mediators, as an angiogenesis factor and a mitogen in skin fibroblasts, respectively, both of which are important biological responses to atopic dermatitis. The SPC level is known to be elevated in atopic dermatitis, resulting from abnormal expression of sphingomyelin (SM) deacylase, accompanied by a deficiency in ceramide. Also, S1P and its receptor, sphingosine-1-phosphate receptor 1 (S1P1) are important targets in treating atopic dermatitis.

**Results:**

In this study, we found a novel antagonist of SPC and S1P1, KRO-105714, by screening 10,000 compounds. To screen the compounds, we used an SPC-induced cell proliferation assay based on a high-throughput screening (HTS) system and a human S1P1 protein-based [^35^S]-GTPγS binding assay. In addition, we confirmed the inhibitory effects of KRO-105714 on atopic dermatitis through related cell-based assays, including a tube formation assay, a cell migration assay, and an ELISA assay on inflammatory cytokines. Finally, we confirmed that KRO-105714 alleviates atopic dermatitis symptoms in a series of mouse models.

**Conclusions:**

Taken together, our data suggest that SPC and S1P1 antagonist KRO-105714 has the potential to alleviate atopic dermatitis.

## Introduction

Atopic dermatitis (eczema) is a common inflammatory skin disease; up to 245 million people globally experienced atopic dermatitis in 2015 [1]. Thus, this disease is one of the major skin diseases threatening public health. Sphingosylphosphorylcholine (SPC) and sphingosine-1-phosphate (S1P) are known as important targets for atopic dermatitis. SPC and S1P are sphingomyelin metabolites generated from sphingomyelin (SM), which is one of the major components in the plasma membranes of eukaryotic cells and regulates cholesterol homeostasis. The sphingomyelin metabolites, S1P, ceramide, sphingosine, and diacylglycerol have important functions in various cellular processes, including cell apoptosis, aging, differentiation, and development [2–5]. SM deacylase hydrolyzes SM to yield SPC rather than the ceramides produced by sphingomyelinase (SMase). Abnormally high expression of SM deacylase in the epidermis of patients with atopic dermatitis causes a ceramide deficiency resulting in atopic dry skin [6, 7]. Okamoto et al. reported that accumulation of SPC in the stratum corneum of patients with atopic dermatitis is related to decreasing ceramide levels in the stratum corneum [8]. SPC was shown to rapidly increase intracellular free calcium ([Ca^2+^]i) and promote a rapid activation of mitogen-activated protein kinase (MAPK) in several different cell types [9]. Because a rise in [Ca^2+^] appears to be an initial and common response to stimulation of proliferation and differentiation in a variety of cell types, SPC is a potent mitogen that promotes DNA synthesis and cellular proliferation to a greater extent than other known growth factors [10, 11]. SPC plays an important role in the inflammatory process by increasing the generation of pro-inflammatory cytokines, such as tumor necrosis factor-α (TNF-α) and interleukin-6 (IL-6) and enhancing wound healing through the release of connective tissue growth factor and adhesion molecules [12–14]. SPC stimulates monocyte chemotactic protein-1 (MCP-1) production through p38 MAPK/JNK- and nuclear factor-κB (NF-κB)/activator protein 1 (AP-1)-dependent pathways in endothelial cells [15]. MCP-1/chemokine (C-C motif) ligand-2 (CCL-2) is a key inflammatory mediator involved in autoimmune pathogenesis [16]. Like MCP-1, SPC is generally thought to be a deleterious factor in skin pathology [17]. For example, SPC elevates intracellular calcium levels and the level of reactive oxygen species in endothelial cells, thereby enhancing their migration [18, 19]. Furthermore, SPC reduced IL-1β and prostaglandin E2 (PGE2) formation through the transforming growth factor (TGF)-β receptor in renal mesangial cells, which contrasts with its pro-inflammatory actions in fibroblasts [20].

Although several studies have attempted to identify a receptor for SPC, no high affinity SPC-specific membrane receptor has been identified, unlike sphingosine 1-phosphate (S1P) and lysophosphatidic acid (LPA) [21]. Shortly after the characterization of ovarian cancer G-protein-coupled receptor 1 (ORG1) and G-protein-coupled receptor 4 (GPR4) as high affinity SPC receptors, evidence was published showing that both of these receptors act as proton-sensing G-protein-coupled receptors (GPCRs) in response to extracellular pH change [22]. Further studies have indicated that the expression of GPR4 reduces extracellular signal-regulated kinase (ERK) activation in a ligand-independent manner [23] and causes SPC to antagonize the pH-induced receptor signaling [24]. Subsequent studies failed to reproduce important features of the original reports characterizing ORG1 and GPR4 as high affinity SPC receptors, and the initial papers were retracted [25]. Some studies showed that G2A is a proton-sensing GPCR antagonized by lysophosphatidylcholine (LPC) [26]. In addition, studies showed that the G2A ligand independently stimulates the accumulation of inositol phosphates and induces apoptosis within the cell [27]. Despite lacking a defined extracellular binding site, a number of studies indicated that SPC-induced effects can occur exclusively via plasma membrane receptors for S1P or LPA [28, 29].

In this study, we found the novel antagonist against both SPC and S1P1 to act as a potential therapeutic agent in treating atopic dermatitis. We performed an SPC-induced proliferation assay using a combinatorial library of 10,000 compounds and found an active compound; KRO-105714 (2,4,5-trisubstituted-1,3-thiazole derivative). We were able to confirm it as a potent S1P1 inhibitor [30]. To demonstrate the therapeutic effects of KRO-105714 on atopic dermatitis, we performed a series of atopic dermatitis-related cell-based assays, including an angiogenesis assay, a cell migration assay and an inflammation cytokine assay. In addition, we confirmed that KRO-105714 alleviated atopic dermatitis symptoms in the mouse models. Taken together, we suggest KRO-105714, an antagonist of SPC and S1P1, as a potential therapeutic agent against atopic dermatitis.

## Results

### SPC-antagonistic effect of a novel compound, KRO-105714 against SPC-induced cell proliferation

**Fig. 1:**
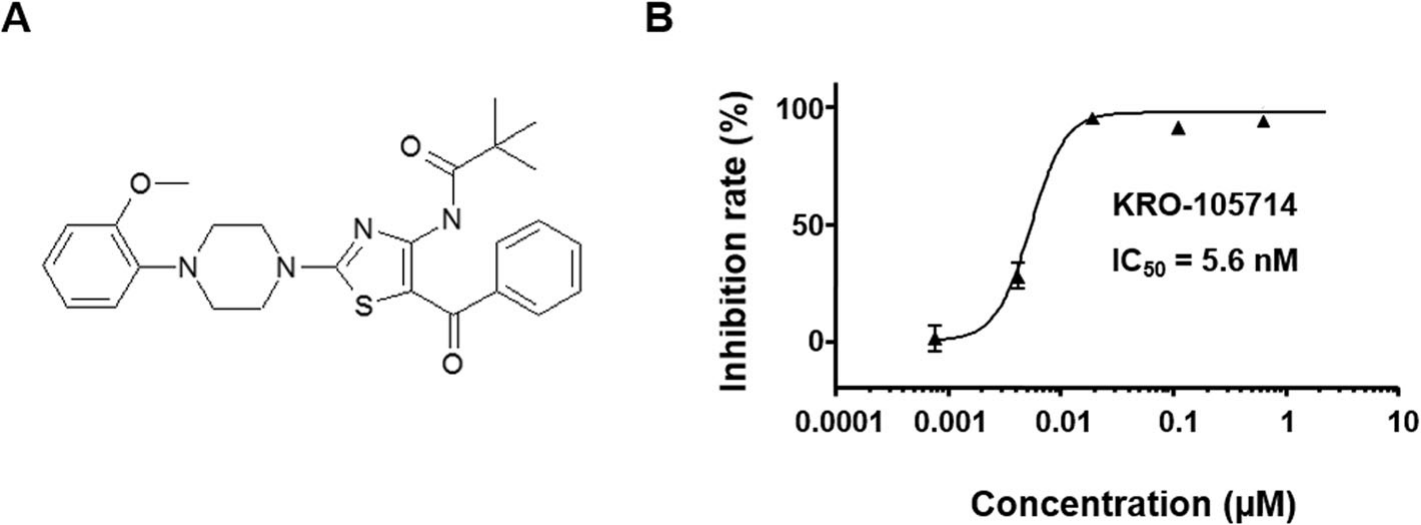
**a** Chemical structure of KRO-105714. **b** The inhibitory effects of KRO-105714 on the proliferation of SPC-induced NIH3T3 cells. The cells were exposed to various concentrations of KRO-105714 for 30 min, and then treated with 10 μM of SPC for 24 h. Then inhibition rates were calculated by comparing the corresponding viability values of the control group. Each sample was assayed in triplicate

In this study, we screened inhibitors of cell proliferation based on SPC-induced cell conditions from a 10,000-compound synthesized by combinatorial methods at the Korea Research Institute of Chemical Technology, using an HTS system. As a result, we acquired 501 compounds that have an inhibitory effect greater than 88% at 2.5 μM as primary hits. The compound (KRO-105714), shown in Fig. 1a, was the most potent compound in SPC-induced cell inhibitory effect; its chemical structure was identified as 2,4,5-trisubstituted-1,3-thiazole. We then examined the effects of KRO-105714 on cell viability. Figure 1b shows KRO-105714 as a potent inhibitor of SPC-induced cell proliferation in NIH3T3 cells. This compound was first reported as an SPC antagonist in this study. Before confirming the SPC-antagonistic effects of KRO-105714, we synthesized KRO-105714 and compared our analytical data with those in the Korean pat. No. 1051078 and U.S. pat. No. 7915405 [30]. Results of ^1^H NMR and ^13^C NMR of our synthesized KRO-105714 compound were shown in Additional file 1: Figure S1. LC-MS (ESI) and HRMS (ESI) were calculated for KRO-105714 (Additional file 1: Figure S2). The SPC antagonist capacity (IC_50_ = 5.6 nM) in Fig. 1b was demonstrated using newly synthesized KRO-105714 as mentioned above.

### Antagonistic activity of KRO-105714 against S1P1 receptor

**Fig. 2:**
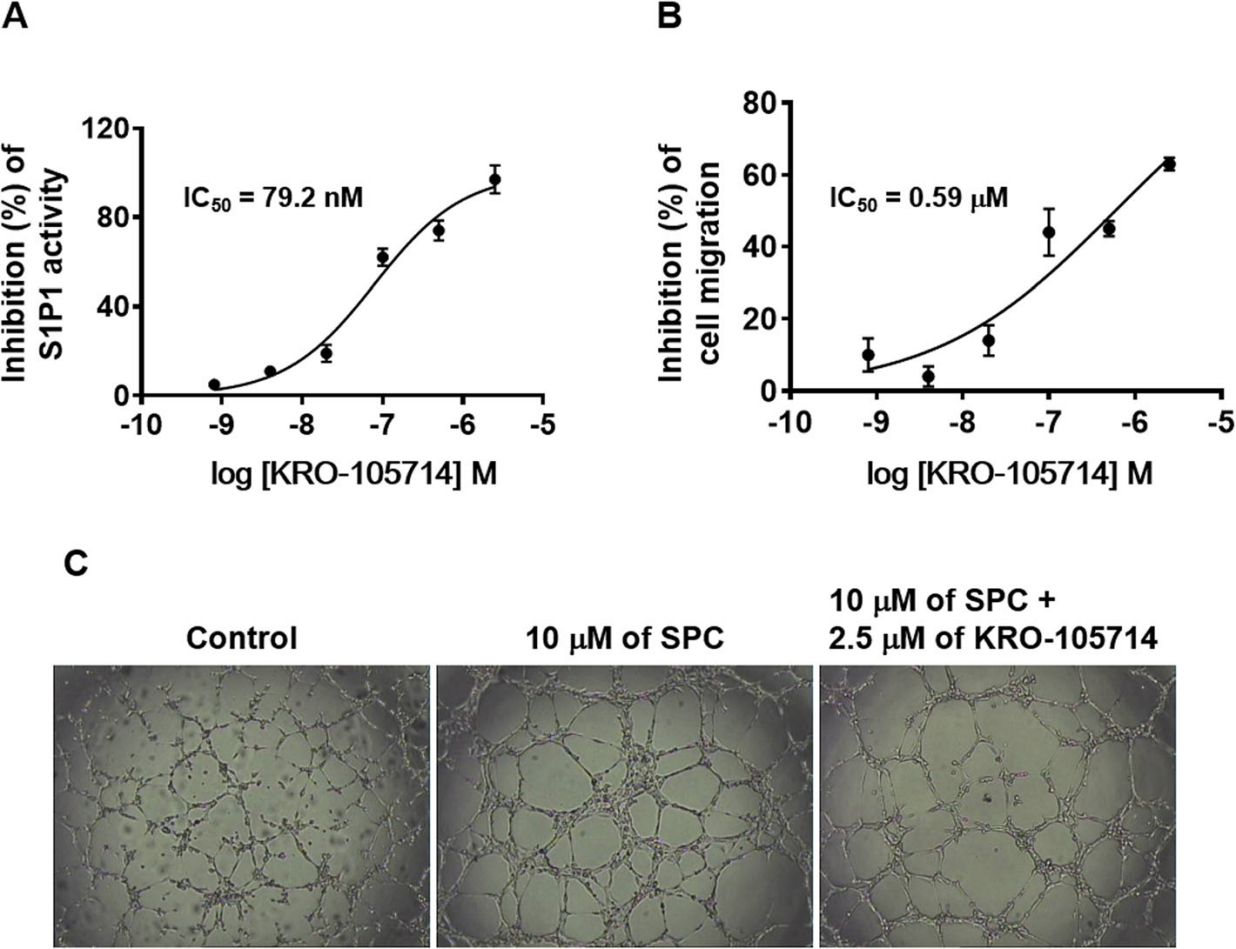
**a** Antagonistic effects of KRO-105714 by SPC (**a**: [^35^S]-GTPγS binding assay, **b**: Migration assay). The HEK-293 cells stably expressing hS1P1 were homogenized with assay buffer and pre-incubated with GTP-binding buffer in the presence or absence of 0.8 nM, 4 nM, 20 nM, 100 nM, 500 nM and 2.5 μM KRO-105714 with 10 μM SPC for 30 min. 1200 Ci/mmol; 0.1 nM [^35^S]-GTPγS was loaded and incubated for 30 min. Nonspecific binding was determined in the presence of 30 μM GTPγS. Each dose of the test compound was assayed in triplicate on pooled samples. **b** Migration assays were performed in Transwell 96-well chambers. SPC (10 μM) was placed in the lower well. HUVECs were harvested and treated with test compounds for 30 min before seeding. The cell suspension was loaded into the upper well. Cells were allowed to migrate for 4 h in a humidified chamber. The cells were fixed and stained with hematoxylin and eosin. Cell migration was quantified by counting the cells that attached to the lower surface of the filter using an optical microscope at X200 magnification. Fifteen fields were counted for each assay. Each dose of the test compound was assayed in triplicate on pooled samples. **c** Tube formation effects of KRO-105714 in HUVECs. Representative photographs of HUVEC tube formation induced by control (vehicle, 0.1% DMSO) (**a**), 10 μM of SPC (**b**), 10 μM of SPC, and 2.5 μM of KRO-105714 are shown from three different independent experiments

We determined whether KRO-105714 affects interactions between SPC and S1P1 receptor. Figure 2a showed that, as expected, treatment with KRO-105714 significantly inhibited the binding of S1P1 and GTP. From the human S1P1-based [^35^S]-GTPγS binding assay, KRO-105714 showed dose-dependent inhibition of GTP binding in lysates of HEK-293 cells expressing S1P1. The IC_50_ value of KRO-105714 in the [^35^S]-GTPγS binding assay was 79.2 nM (Fig. 2a). These results suggest that KRO-105714 acts as an antagonist of S1P1.

### Inhibitory effect of KRO-105714 on SPC-induced migration in endothelial cells and SPC-induced endothelial tube formation

Increased angiogenesis is one of the most critical characteristics in atopic dermatitis. SPC is also a well-known material for its pro-angiogenetic capacity. Thus, we wondered whether KRO-105714 had an inhibitory effect on SPC-mediated angiogenesis and examined migration to investigate the effect of KRO-105714 on SPC-induced endothelial cell migration. First, we found that SPC-stimulated chemotactic migration of HUVECs was dose-dependently inhibited by KRO-105714, and the IC_50_ in the migration assay was measured as 0.59 μM (Fig. 2b). Then, we examined the anti-SPC effect of KRO-105714 on SPC-induced tube formation in HUVECs. Figure 2c shows that 2.5 μM of KRO-105714 antagonized SPC-induced tube formation (Fig. 2c).

### Inhibitory effects of KRO-105714 on SPC-induced Th2 cytokine production

**Fig. 3:**
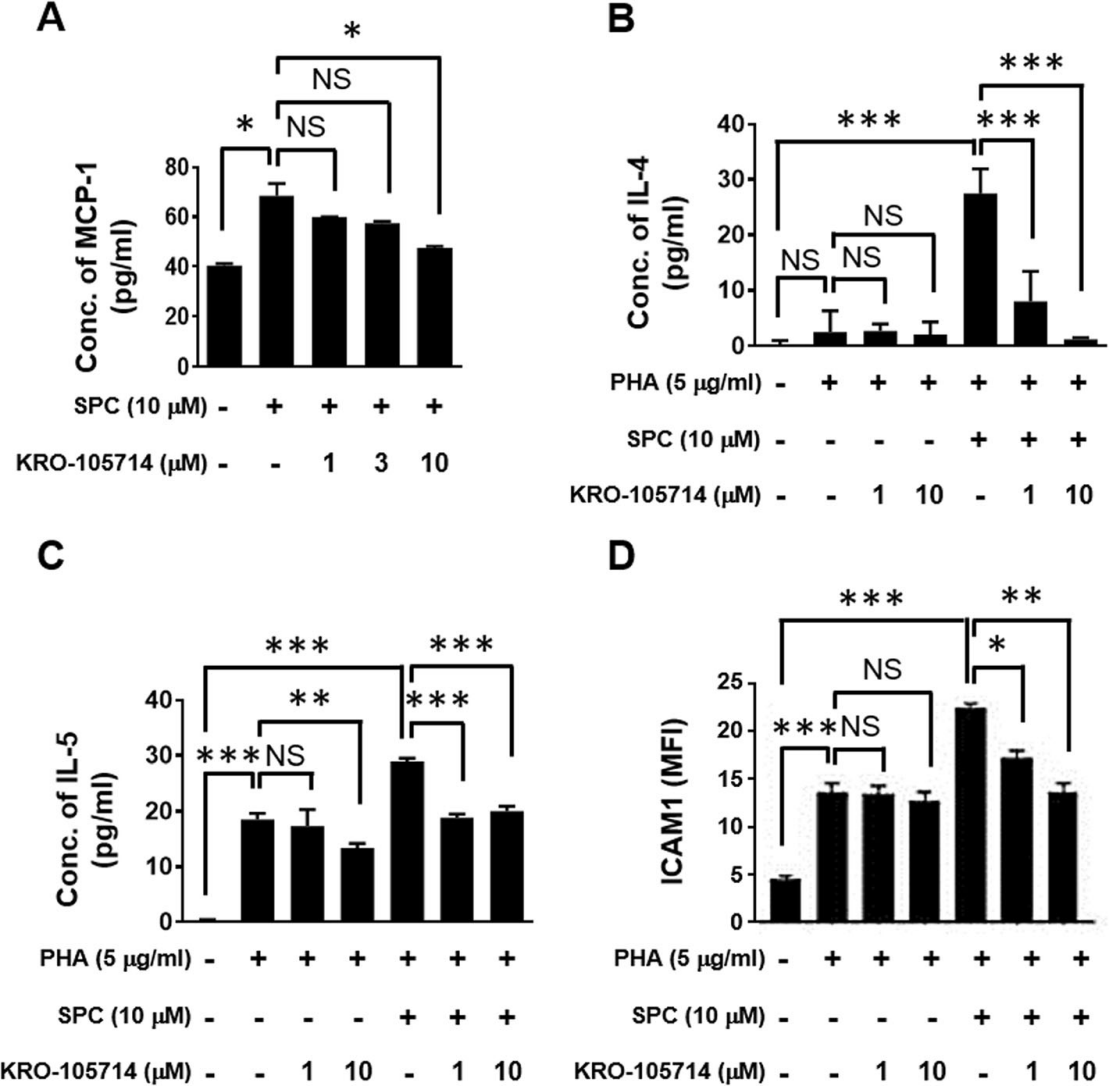
**a** Effects of KRO-105714 on chemokine levels in stimulator-induced RAW 264.7 cells. The RAW 264.7 cells were seeded in 96-well plates at a density of 2 × 10^4^ cells/well and pre-treated with various concentrations (1, 3, 10 μM) of KRO-105714 for 30 min and then treated with 10 μM of SPC for 24 h. Expression levels of MCP-1 were measured using ELISA. Each dose of the test compound was assayed in triplicate and the assays were repeated three times. Data are shown as mean ± standard deviation (SD). *P < 0.05, ** *P* < 0.01 and *** *P* < 0.001 is the significance level compared to the SPC treated group. **b**-**c** Inhibitory effects of KRO-105714 on the expression of pro-inflammatory cytokines and adhesion molecule. The cytokines used were PBMCs isolated from the blood of healthy individual donors. The human PBMCs were seeded in 96-well plates at a density of 5 × 10^5^ cells/well and pre-treated with concentrations (1 and 10 μM) of KRO-105714 for 30 min and then treated with 10 μM of SPC and 5 μg/ml of PHA for 24 h (**b**) of 72 h (**c**). IL-4 (**b**), and IL-5 (**c**) levels were measured using ELISA. Each dose of the test compound was assayed in triplicate. ICAM-1 expression level of PBMCs (**d**) was measured using flow cytometry. MFI means “mean fluorescence intensity”. Data are shown as mean ± standard deviation (SD). *P < 0.05, ** *P* < 0.01 and *** *P* < 0.001 versus compound-untreated cells (ANOVA multiple comparison)

To evaluate the effectiveness of KRO-105714 on pro-inflammatory chemokine and cytokine production from macrophages in an SPC-induced inflammatory condition, we treated the RAW 264.7 cells and human PBMCs with KRO-105714 in the presence or absence of SPC (Fig. 3). Cell supernatant was collected and analyzed using ELISA kits. As expected, KRO-105714 inhibited SPC-induced MCP-1 release in RAW 264.7 cells (Fig. 3a). Also, we investigated the inhibitory effects of KRO-105714 on Th2 cytokines: IL-4 and IL-5, which are important for allergic-responses. Human PBMCs treated with KRO-105714 (1 and 10 μM) were exposed to PHA with/without SPC, to induce allergic-response conditions for 24 h or 72 h. Then, the supernatant was collected for measurement of the cytokines. As shown in Fig. 3, we confirmed that KRO-105714 suppressed IL-4 and IL-5, triggered by SPC in the presence of PHA in PBMCs. As a result, KRO-105714 was found to have an antagonistic effect on the SPC-induced production of Th2 cytokines IL-4 and IL-5 from PBMCs in a dose-dependent manner. Also, ICAM-1, an adhesion molecule known to be induced in atopic dermatitis patients than normal individuals, were down-regulated by KRO-105714 compound in SPC and PHA co-treated PBMCs (Fig. 3d) [31].

### Effects of KRO-105714 in multiple in vivo atopic dermatitis models

**Fig. 4:**
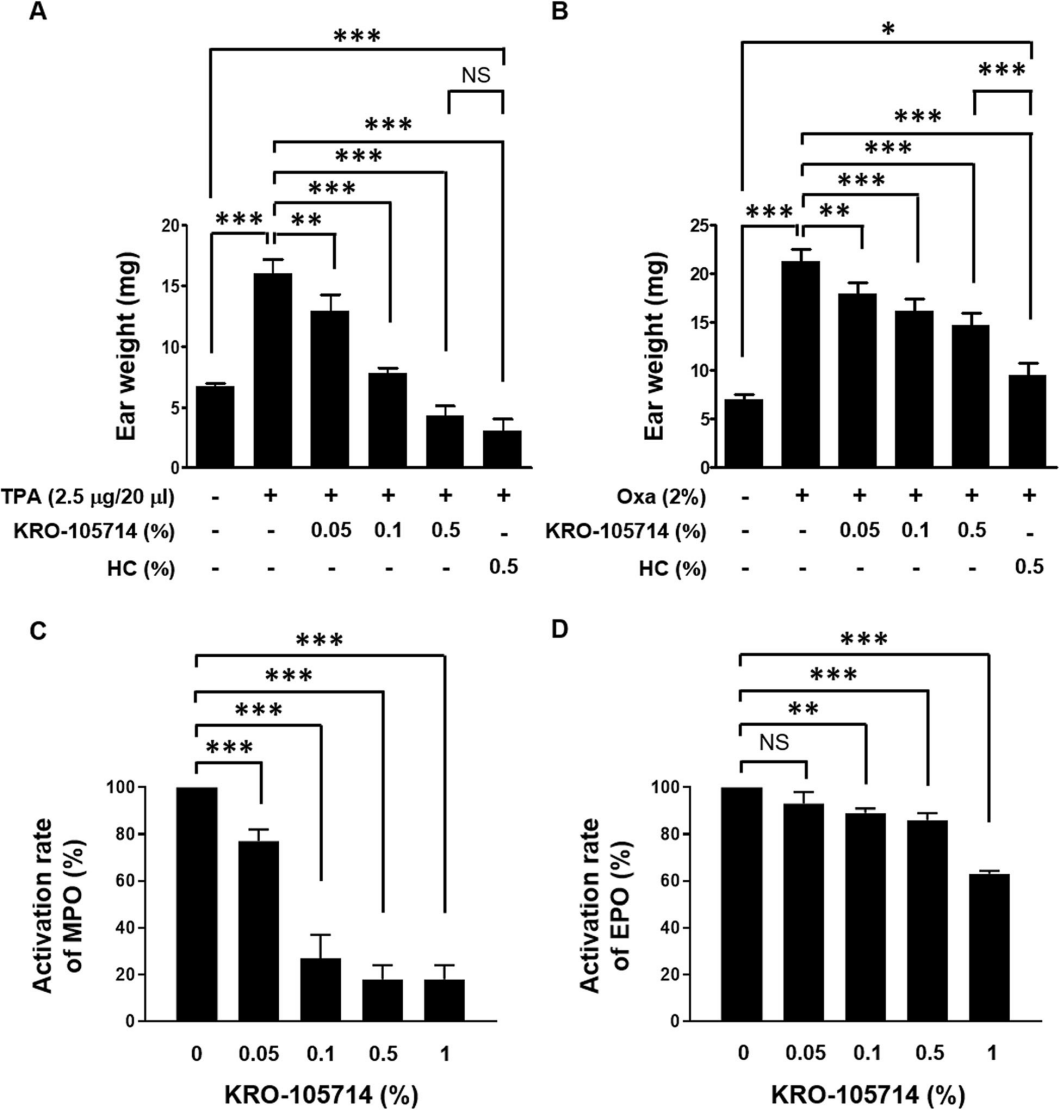
Alleviated effects of skin inflammation in ear edema models (**a**, **b**: measurement of ear weight, **c**: myeloperoxidase assay, **d**: eosinophil assay). KRO-105714 solution was applied to the ears of ICR mice twice in 15 min and 6 h after application of TPA (**a**, **c**) or in 0 h and 6 h after application of oxazolone (**b**, **d**). **a**, **b** After 24 h, a whole ear weight was measured using an electronic balance. Then, expression levels of MPO (**c**) or EPO (**d**) in ear skin were analyzed. Data are shown as mean ± standard deviation (SD). NS: not significant; NS: not significant; *P < 0.05, ** *P* < 0.01 and *** *P* < 0.001 versus compound-untreated cells (ANOVA multiple comparison)

**Fig. 5:**
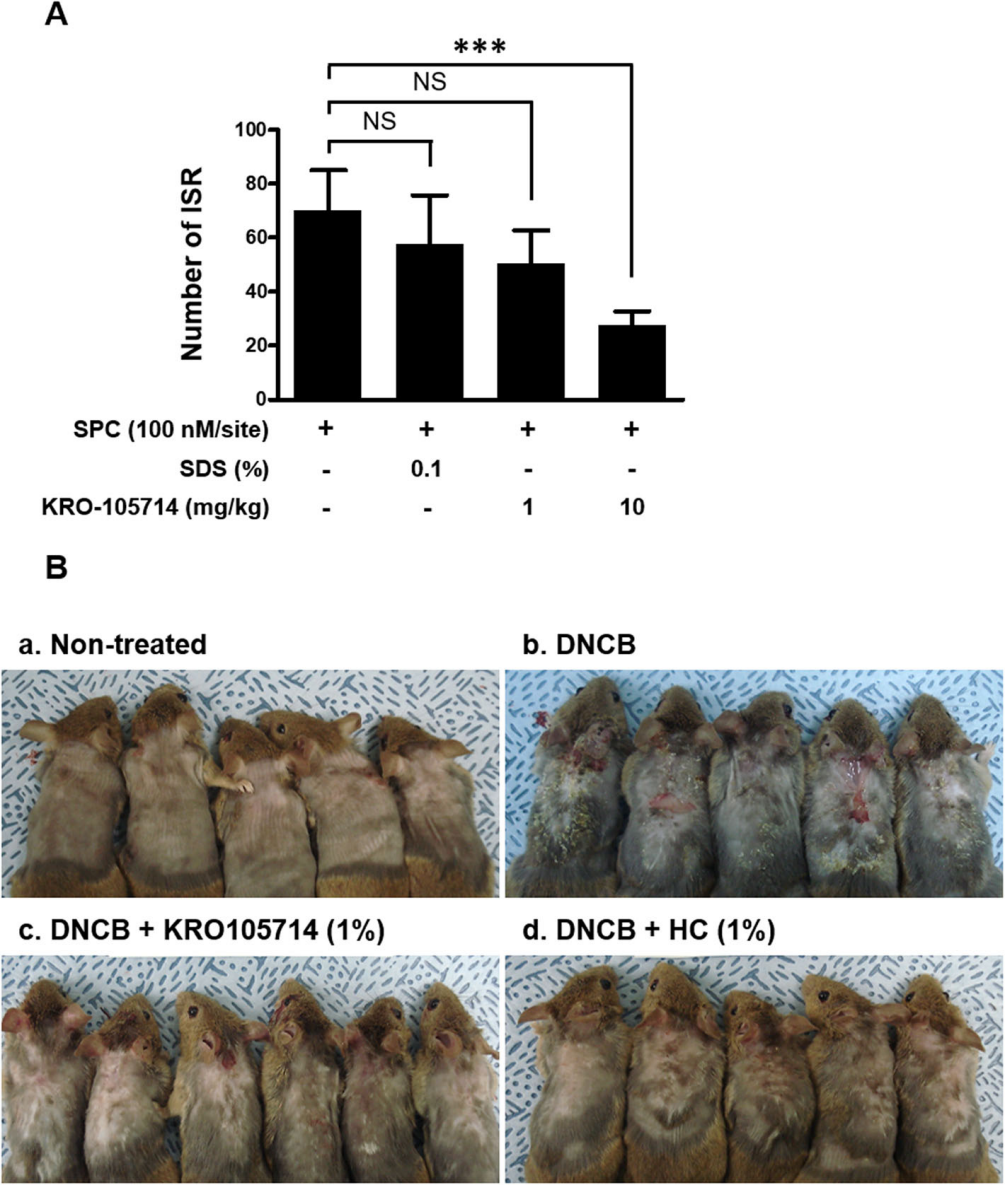
Effects of KRO-105714 treatment on itch-scratch response (ISR) and DNCB-induced atopic dermatitis in animal models. **a** itch-scratch response (ISR) of evaluation of scratching behavior mouse model. To measure itch-related behavior induced by SPC, KRO-105714 was administered to ICR mice (8 weeks, male, *n* = 5 per group) via oral administration of 1 and 10 mg/kg KRO-105714 with the SPC application (100 nM/site), bouts of scratching were counted for 30 min. Data are shown as mean ± standard deviation (SD). *P < 0.05, and ** *P* < 0.01 versus mice with vehicle (*n* = 5) (ANOVA multiple comparison). **b** Images of skin lesions from the groups of mice were taken on the last day of the experiment (a; Non-treated, b; 0.2% DNCB, c; 0.2% DNCB/1% KRO-105714, d; 0.2% DNCB/1% hydrocortisone). Representative images from five different mice per group are shown

In vivo efficacy of KRO-105714 was determined in TPA-induced atopic dermatitis ear model. Hydrocortisone (HC), a potent topical corticosteroid, was chosen as the reference compound [32]. As shown in Fig. 4a, KRO-105714 reduced ear weights of TPA-induced atopic dermatitis mice. TPA treatment increased ear thickness in this mouse model and KRO-105714 applied on the ear skin significantly reduced ear swelling symptoms and the reduction efficacy was dose-dependent. 0.5% of KRO-105714 exhibited a similar anti-inflammatory effect to 0.5% HC. KRO-105714 also showed a therapeutic effect in an oxazolone-induced mouse model, although the efficacy was weaker than HC (Fig. 4b). To understand the therapeutic mechanism associated with KRO-105714 in these dermatitis models, we analyzed MPO and EPO activities, which are well-known for their critical roles in dermatitis. As a result, KRO-105714 significantly reduced MPO activity in TPA-induced dermatitis tissues. Specifically, KRO-105714 showed an inhibition rate of about 80% when applied at 0.5 and 1.0% in the TPA model (Fig. 4c). In addition, KRO-105714 inhibited the activity of EPO at about 40% in the oxazolone-induced mouse model (Fig. 4d). Furthermore, to investigate the effect of KRO-105714 on scratching behavior, which is one of critical indicates for atopic dermatitis, we used the DNCB-induced atopic dermatitis mouse model and measured itch-scratch response (ISR). To evaluate the effectiveness of KRO-105714 on ISR of the DNCB-induced atopic dermatitis-like skin lesions, the mice were treated with 1% KRO-105714 and 0.2% DNCB daily for 4 days. As shown in Fig. 5a, oral administration of KRO-105714 reduced SPC-induced ISR in a dose-dependent manner compared to the untreated group in the DNCB-induced atopic dermatitis mice. Also, repeated DNCB application significantly increased multiple atopic dermatitis symptoms, such as hemorrhage edema, scarring, dryness, and erosion in NC/Nga mice (Fig. 5b). Only DNCB treated mice showed severe hemorrhage edema, scarring, dryness, and erosion symptoms. However, KRO-105714 at 1% with DNCB treated mice showed much less hemorrhage edema, scarring, dryness, and erosion symptoms compared to only DNCB treated mice. Also, the alleviating effect on atopic dermatitis mice by KRO-105714 was a similar level to the HC-treated with DNCB group.

## Discussion

SPC and S1P/S1P1 are known as important players in the pathological pathways of atopic dermatitis. In previous reports [6–8], the concentration of SPC is increased in atopic dermatitis lesions of clinical patients and a concomitant decrease in ceramide levels is regarded as a pathogenic factor, because Sphingomyelin (SM) is metabolized to ceramide by sphingomyelinase (SMase) or alternatively metabolized SPC by SM deacylase [33, 34]. SPC functions as a signal transducer in various cellular processes, such as proliferation, differentiation, migration and apoptosis, and works as an important mediator of atopic dermatitis [2, 10]. Moreover, SPC is known as a stimulation factor for multiple immune cells, such as monocytes and macrophages, to amplify inflammation in many inflammatory diseases [34]. Thus, the level of SPC in skin inflammatory lesions might be an indicator of atopic dermatitis. However, so far, no SPC receptor has been clearly identified. Thus, the use of an SPC antagonist is the only method to block the SPC induced signal pathway. Thus, our study, which focused on finding SPC antagonists, considered the importance of SPC in atopic dermatitis pathology. In addition, S1P is metabolized from SM and functions as a pro-inflammatory mediator and angiogenesis factor, which are biological responses related to atopic dermatitis [35, 36]. Thus, S1P or its receptor, S1P1 should also be therapeutic targets for atopic dermatitis. Our SPC and S1P1 dual inhibitory compound, KRO-105714 should therefore be a potent candidate molecule for atopic dermatitis.

In this study, we identified KRO-105714 as an SPC antagonist and inhibitor of the S1P receptor, S1P1, by screening inhibitors from a 10,000-compound library based on the SPC-induced cell proliferation and a human S1P1 protein-based [^35^S]-GTPγS binding assay. KRO-105714 showed potent SPC inhibitory (IC_50_ = 5.6 nM) and antagonistic (IC_50_ = 79.2 nM) activities in GTP binding assay (Fig. 2a and b). Increased angiogenesis, fibrosis, and infiltration of immune cells are critical characteristics of atopic dermatitis. In a previous report [37], researchers showed that SPC has pathological vasculogenic and fibrogenic activities in atopic dermatitis. *Piao* et al. explained that SPC can induce endothelial cell sprouting in an in vitro angiogenesis model and increased tube-like formation in an in vivo wound healing model [38]. Based on those previous reports, SPC’s pathological angiogenic power is an important therapeutic target in atopic dermatitis. As we expected, KRO-105714 showed a potent inhibition on the SPC-induced HUVEC tube formation and endothelial cell migration (IC_50_ = 0.59 μM) (Fig. 2c) indicating KRO-105714 an anti-angiogenic compound.

Equally important is the cytokine blocking effect of SPC in skin diseases, which has been suggested to play a role in the inflammatory processes of the epidermis through up-regulation of monocyte chemotactic protein-1 (MCP-1) [6]. MCP-1 is a well-known potent inflammatory chemokine which exacerbates inflammatory dermatitis by recruiting inflammatory immune cells such as monocytes, macrophages, and neutrophils [39]. Because MCP-1 is a potent chemotactic factor that triggers the infiltration of monocytes/macrophages into inflammatory sites [15], expression of MCP-1 is an indicator for many inflammation-associated pathological states, such as dermatitis [6], rheumatoid arthritis, atherosclerosis [40], diabetic nephropathy [39], lung inflammation [40] and cancer [41]. It has been reported that the administration of MCP-1 inhibitors inhibits macrophage accumulation into inflammatory lesions and improves disease outcomes [42]. Based on these reports, SPC should be a triggering factor to increase expression of MCP-1 from mouse monocytes and human PBMCs to increase infiltration of immune cells. Thus, the inhibitory effect of KRO-105714 on expression of MCP-1 would contribute in reducing inflammation in the dermatitis lesions.

In atopic dermatitis, Th2 cytokines such as IL-4 and IL-5 are known to have an important function in amplifying allergic inflammation in skin lesions [43]. In this study, we found that SPC strongly trigger secretion of Th-2 cytokines (IL-4 and IL-5) relating to allergic reactions from PBMCs (Fig. 3b and c). As previously reported, IL-4 and IL-5 mediate the secretion of IgE in B cells [44]. These previous studies support the role of SPC as an allergic effector in atopic dermatitis [6–8, 29]. Thus, we confirmed that KRO-105714 inhibits an SPC-induced secretion of Th-2 cytokines. Actually, this study is the first report to show SPC co-treatment with PHA enhanced IL-4 and IL-5 induction from PBMCs. Because the role of SPC in production of IL-4 and IL-5 is important to understand allergic responses, this should be further investigated to understand the underlying mechanisms. For in vivo experiment, we still need to identify which is the proper animal model for this finding. There is no previous report that SPC induced IL-4 and IL-5 in vivo model yet. Also, these allergic response related that SPC induced Th2 cytokine production should be further studied to clearly understand the mechanism of KRO-105714 on SPC related to atopic dermatitis.

Topical application of TPA and oxazolone is a valid model to screen potential therapeutic agents for treatment of inflammatory dermatitis [45, 46]. The measurements of MPO activity confirm that KRO-105714 could inhibit neutrophil level in inflammatory lesions of a TPA-induced mouse model [47] (Fig. 4c). Increased infiltrated eosinophils in atopic dermatitis lesions is a well-known feature of most patients with atopic dermatitis, and T cell activation by antigen-presenting cells leads to the production of Th2 cytokines that support eosinophil functions [48]. As we discussed, reduction of MCP-1 expression levels by KRO-150714 might be the factor to reduce MPO and EPO, because neutrophil and eosinophil also used the chemokine, MCP-1 [49]. Further study is required to understand this MCP-1 related mechanism for reduced MPO and EPO. In this study, to determine whether the confirmed activity of KRO-105714 on anti-inflammation correlates in vivo in three different mouse models, we performed repeated applications of TPA and oxazolone to sensitize mice for induction of atopic dermatitis associated symptoms. In both the TPA and oxazolone induced dermatitis models, KRO-105714 reduced ear weights and MPO and EPO activity (Fig. 4). Also, in a scratch behavior experiment using a DNCB-induced dermatitis mouse model, KRO-105714 markedly inhibited SPC-induced ISRs when orally administered to mice (Fig. 5a and b). Similarly, KRO-105714 caused recovery in the atopic dermatitis symptoms such as hemorrhage edema, scarring, dryness, and erosion from DNCB-induced skin inflammatory response in NC/Nga mice (Fig. 5c).

As we demonstrated, KRO-105714 showed potent anti-allergic activity in both the in vitro and in vivo assays, implying its potential value as a novel therapeutic for atopic dermatitis. For evaluation of KRO-105714 as a drug candidate, we testified its important toxicities such as hepatotoxicity (Additional file 1: Figure S3) and hERG (Additional file 1: Table S1). After oral administration of KRO-105714 compound (250 mg per kilogram of mouse weight and 500 mg per kilogram of mouse weight) did not show any effect on alanine aminotransferase (ALT) activity from blood plasma of mice (Additional file 1: Figure S3). KRO-105714 did not inhibit CYP450, indicating it had no drug-induced hepatotoxicity (1A2: > 10 μM, 2C9: > 10 μM, 2C19: > 10 μM, 2D6: > 10 μM, 3A4: > 10 μM (IC_50_)) (Additional file 1: Table S1A). KRO-105714 also had no cardiotoxicity in the hERG potassium channel binding assay (IC_50_ > 10 μM) (Additional file 1: Table S1B).

## Conclusions

Taken all together, this study demonstrates that KRO-105714 acts as a dual inhibitor of SPC and S1P1 to efficiently alleviate atopic dermatitis symptoms in vivo*/*in vitro through reduction of atopic dermatitis-related chemokines and cytokines, angiogenesis and monocyte/macrophage infiltration. Although further study is needed to delineate the detailed signal pathways underlying the mode of action of KRO-105714, our results strongly suggest that KRO-105714, a novel SPC and S1P1 antagonist, is a potential therapeutic reagent with low toxicity for atopic dermatitis.

## Methods

### Reagents

SPC was purchased from Matreya Inc. (Pleasant Gap, USA). Lipopolysaccharide (LPS) and lectin from *Phaseolus vulgaris* (PHA) were provided by Sigma Chemical Co. (St. Louis, MO, USA). 1-Chloro-2,4-dinitrobenzene (DNCB), hydrocortisone (HC), 12-O-tetradecanoylphorbol-13-acetate (TPA) and 4-ethoxymethylene-2-phenyl-2-oxazolin-5-one (Oxazolone) were purchased from Sigma Chemical Co. (St. Louis, MO, USA). Other organic solvents and chemicals were of analytical grade or complied with established standards for cell culture experiments.

### Cell culture and treatments

NIH3T3 (murine fibroblast) cells and HEK-293 (human embryonic kidney) cells were obtained from American Type Culture Collection (ATCC, Manassas, VA, USA) and were maintained in DMEM (Gibco, BRL, Grand Island, NY, USA), supplemented with 10% fetal bovine serum (FBS) and 100 U/ml of penicillin-streptomycin, at 37 °C in 95% humidity and 5% CO_2_ atmosphere. Human umbilical vein endothelial cells (HUVECs) were obtained from Bio4You (Seoul, South Korea) and were maintained in M199 (Gibco, BRL, Grand Island, NY), supplemented with 20% FBS, basic fibroblast growth factor (bFGF) (R&D Systems, Minneapolis, MN, USA), heparin, and 100 U/ml of penicillin-streptomycin at 37 °C in 95% humidity and a 5% CO_2_ atmosphere. Raw 264.7 (mouse macrophage) cells were obtained from the Korean Cell Line Bank (KCLB, Seoul, South Korea) and were maintained in DMEM supplemented with 10% FBS and 100 U/ml of penicillin-streptomycin at 37 °C in 95% humidity and a 5% CO_2_ atmosphere. Human peripheral blood mononuclear cells (PBMCs) were isolated from the blood of healthy individual donors by density-gradient centrifugation through a Ficoll-Paque (GE Healthcare, Chicago, IL, USA) gradient. The isolated human PBMCs were cultured in RPMI1640 (Gibco, BRL, Grand Island, NY, USA) and supplemented with 10% FBS and 100 U/ml of penicillin-streptomycin.

### Human peripheral blood

Peripheral blood from healthy donors was obtained from the Red Cross Blood Center. We followed the guidelines of the Red Cross, and all of the methods and protocols used in this study with human peripheral blood were approved by the Institutional Review Board of the Red Cross. Based on the Red Cross guidelines, informed consent for study participation was obtained and donor information could not be provided. For preparation of Peripheral blood mononuclear cells (PBMC), we isolated PBMCs using ficoll-gradient centrifugation.

### High-throughput screening (HTS) for SPC-induced cell inhibition assay

NIH3T3 cells were seeded in 96-well plates at a density of 5 × 10^3^ cells/well and stabilized overnight. The cells were treated with test compounds at 25 nM, 50 nM, 0.25 μM, and 2.5 μM for 30 min, following which10 μM of SPC was added. The cells were incubated for 24 h and 0.5 μCi/well of [3H]-thymidine was loaded and further incubated for 24 h. The medium was aspirated, and the cells were thoroughly washed three times with PBS. Subsequently, a scintillation cocktail was added, and the radioisotope that had been incorporated into the cells was quantified using a MicroBeta® TriLux (PerkinElmer, Waltham, MA, USA). The automated screening assay was performed in an HTS setup using Biomek FX and ORCA robot system (Beckman Coulter, Pasadena, CA, USA).

### [^35^S]-GTPγS binding assay

HEK-293 cells stably expressing hS1P1 were homogenized in an assay buffer (50 mM HEPES buffer, pH 7.5, containing 100 mM NaCl, 5 mM MgCl_2_, 20 μM GDP) and centrifuged at 45,000×g for 20 min at 4 °C. Aliquots of pellets (5 μg) were pre-incubated with GTP-binding buffer (50 mM HEPES buffer, pH 7.5, containing 100 mM NaCl, 5 mM MgCl_2_, 20 μM GDP, 0.1% BSA, 20 μg/ml saponin) in the presence of the KRO-105714 (0.8 nM, 4 nM, 20 nM, 100 nM, 500 nM and 2.5 μM) for 30 min at 30 °C. [^35^S]-GTPγS (1200 Ci/mmol; 0.1 nM) was loaded and incubated for 30 min at 30 °C. The reaction was then terminated by rapid filtration under vacuum through a UniFilter GF/C microplate, then scintillant is melted onto the filter. The filter-bound radioactivity was counted using a MicroBeta® Trilux. Nonspecific binding (NSB) was determined in the presence of 30 μM GTPγS. NSB was subtracted from each total binding of the indicated concentration of KRO-105714 and the specific binding (SB) was analyzed using four parameter nonlinear regression with GraphPad Prism to yield inhibition values (IC_50_).

### Migration assay

Migration assays were performed in Transwell 96-well chambers with 8-μm polycarbonate membrane filters (Becton Dickinson Labware, Franklin Lakes, NJ). SPC (10 μM), prepared in RPMI1640 with 1% FBS, was placed in the lower well. HUVECs were harvested at a density of 2.5 × 10^5^ cells/ml and treated with compounds at 0.8 nM, 4 nM, 20 nM, 100 nM, 500 nM, and 2.5uM for 30 min before seeding. The cell suspension (100 μl) was loaded into the upper well. Cells were allowed to migrate for 4 h in a humidified chamber at 37 °C with 5% CO_2_. The cells were fixed and stained with hematoxylin and eosin. Migration was quantified by counting the cells that had attached to the lower surface of the filter using an optical microscope at X200 magnification. Fifteen fields were counted for each assay.

### Tube formation assay

Matrigel (10 mg protein/ml, Clontech, MA, USA; 40 μl) was pipetted into a 96-well culture plate and polymerized for 1 h at 37 °C. HUVECs were harvested after trypsin-EDTA treatment, re-suspended in M199 and then plated onto a layer of Matrigel at a density of 2 × 10^4^ cells/well, followed by the addition of 10uM SPC in the absence or presence of 2.5uM KRO-105714. After the Matrigel cultures were incubated at 37 °C for 24 h, the cultures were photographed (X40).

### Elisa

Raw 264.7 cells and human PBMCs were seeded in 96-well plates and pre-treated with of 1, 3, and 10 μM KRO-105714. After incubating for 30 min, raw 264.7 cells were added with 10 μM SPC and PBMCs were added with 5 μg/ml PHA in the absence or presence of 10 μM SPC. Supernatants were collected over a time course after the addition of stimulators, and analyzed using ELISA kits in accordance with the manufacturers’ instructions. ELISA kits (MCP-1, IL-4 and IL-5) were purchased from BD Biosciences (Franklin Lakes, NJ). We used normalization method using standard curve with recombinant MCP-1, IL-4 and IL-5 provided from vendor.

### Animals

All the animals were purchased from Orient Bio (Seoul, South Korea) and acclimated under specific pathogen-free conditions in an animal facility for at least a week before use. The mice were housed in a temperature (23 ± 2 °C) and relative humidity (40–60%) controlled room. Lighting was adjusted automatically at a cycle of 12 h light and 12 h dark. For sampling tissues from mice, the animals were sacrificed by cervical dislocation. All the animal protocols used in this study were approved by the Korea Research Institute of Chemical Technology (KRICT) Animal Care and Use Committee.

### Mouse ear edema inflammation model using TPA

Dermatitis was induced by topical application of TPA (2.5 μg/20 μl in acetone) to the ears of Crl:CD1 (ICR) mice (8 weeks, male). Various concentrations (0.01, 0.05, 0.1, 0.5%) of KRO-105714 and 0.5% of hydrocortisone (HC) were topically applied to the ear twice: 15 min and 6 h after TPA treatment. The control group was treated with the vehicle alone (in acetone). Twenty-four hours after TPA application, the mice were sacrificed by cervical dislocation, and samples were collected with a 6 mm diameter metal punch and weighed. Tissue samples were assessed biochemically with the neutrophil marker enzyme myeloperoxidase (MPO) [29]. To measure MPO activity, homogenized tissue were freeze-thawed 3 times, after which sonication was repeated. Suspensions were then centrifuged at 40,000×g for 15 min and the resulting supernatants assayed. One hundred microliter of the ear tissue supernatants to be measured was mixed with 2.9 ml of 50 mM phosphate buffer (pH 6.0) containing 0.167 mg/ml o-dianisidine dihydrochloride and 0.0005% hydrogen peroxide. Chlorination activity of MPO was evaluated with 3′-(p-aminophenyl) fluorescein (APF) and 3′-(p-hydroxyphenyl) fluorescein (HPF, both from Cayman Chemicals, Ann Arbor, MI) 20 μl sample were combined with 40 μl 2 μM H_2_O_2_ and 40 μl 20 μM APF or HPF (in 1% methyl acetate). Fluorescence was measured at 499 nm excitation and 515 nm emission after 30 min. HPF fluorescence values were subtracted from APF in order to calculate MPO chlorination activity.

### Mouse ear edema inflammation model using oxazolone

On study day 0 and 1, Balb/c mice (8 weeks, male) were sensitized with aliquots of 50 μl of a 2% oxazolone solution epicutaneously on their shaved abdomens. On day 6, the right ear of each mouse was challenged with 2% oxazolone solution. Left ears were painted on both sides with an ethanol/acetone mixture. The right ear of each mouse was treated with the indicated concentration (0.05, 0.01, 0.1, 0.5%) of KRO-105714 and 0.5% HC twice (10 μl on the front and 10 μl on the back) at both 0 h and 6 h after the oxazolone-challenge. The control group was treated with the vehicle alone (in acetone). After 24 h, the mice were sacrificed, and ear samples were collected using a 6 mm diameter metal punch and weighed. Tissue samples were assessed biochemically with the eosinophil marker enzyme, eosinophil-peroxidase (EPO). To measure EPO activity, aliquots of the ear tissue supernatants and 100 μl of 50 mM Tris-HCl buffer (pH 8.0) containing 0.1% Triton X-100, 1 mM ο-phenylenediamine and 500 μM hydrogen peroxide were incubated for 30 min at room temperature. The reaction was stopped by adding 50 μl of 2 M sulfuric acid and the absorbance at 490 nM was measured using a microplate reader.

### Evaluation of scratching behavior

To measure itch-related behavior induced by SPC, KRO-105714 was administered to ICR mice (8 weeks, male) via oral administration of 1 and 10 mg/kg KRO-105714 in 200 μl volume of 10% DMSO in PBS per mouse using flexible oral gavage needle (Instech Laboratories, Plymouth Meeting, PA, USA) 30 min before the SPC challenge. After SPC application (100 nM/site), bouts of scratching were counted for 30 min. Mouse scratching behavior was automatically detected and objectively evaluated with a MicroAct apparatus (Neuroscience Inc.). A small magnet (1 mm in diameter, 3 mm long, coated with Teflon) was inserted subcutaneously into both hind paws under ether anesthesia before the start of the experiment. Scratching of the rostral back and biting of the caudal back were observed; scratching movements by the hind paw were defined as a scratching bout, which ended when the mouse either licked its hind paw or placed its hind paw back on the floor. A series of one or more biting movements was counted as one episode, which ended when the rat returned to the straight-forward position.

### DNCB-induced contact dermatitis

To induce dermatitis, NC/Nga mice (5 weeks, male) were sensitized by painting 100 μl of 1% DNCB solution (in acetone:olive oil = 4:1, v/v) onto the shaved back skin (2 × 4 cm). After 4 days, mice were first immunized by painting 150 μl of 0.2% DNCB solution three times per 7-day period for 3 weeks. Twenty-five days later, the animals were challenged with 150 μl of a mixture of 1% of KRO-105714 or 1% of hydrocortisone (HC) with 0.2% DNCB solution in polyethylene glycol:ethanol (7:3, v/v), once daily for 4 days.

### Flow cytometry

Human PBMCs were seeded in 96-well plates and pre-treated with 1 and 10 μM of KRO-105714. After incubating for 30 min, these cells were treated with 10 μM SPC and/or 5 μg/ml PHA. PBMCs were analyzed by flow cytometry using 1 μg/ml of anti-CD45-APC (BioLegend, San Diego, CA, USA), anti-ICAM-1 (BioLegend) and anti-CD44 antibodies in phosphate-buffered saline (PBS) plus 1% fetal bovine serum (FBS) on ice for 10 min. Cells were then washed and analysed. Staining data were collected using a FACSCanto II Cytometer (BD Biosciences). To set the gates for defining positive and negative cells at 5000 cells in multicolor staining, samples were stained with a mixture of all antibodies [50].

### Statistical analysis

ANOVA multiple comparisons were performed to analysis differences among more two groups. Statistical analyses were performed using the Prism software package (GraphPad Software, San Diego, CA, USA). Differences with *P* values of 0.05 or less were considered significant.

## Supplementary information

**Additional file 1: Supplementary data.** A novel sphingosylphosphorylcholine and sphingosine-1-phosphate receptor 1 antagonist, KRO-105714, for alleviating atopic dermatitis. **Supplementary figure 1.** NMR results of KRO-105714 compound. **Supplementary figure 2.** LC-MS/HRMS result of KRO-105714 compound. **Supplementary figure 3.** Liver toxicity and alanine aminotransferase activity (ALT) of KRO-105714. **Supplementary table 1.** Hematotoxicity and hERG potassium channel binding assay of KRO-105714.

## Data Availability

The datasets used /or analyzed during the current study are available from the corresponding author on reasonable request.

## Abbreviations

Eczema: Atopic dermatitis
SPC: Sphingosylphosphorylcholine
SM: Sphingomyelin
TNF-α: Tumor necrosis factor-α
IL-6: Interleukin-6
NF-κB: Nuclear factor-κB
AP-1: Activator protein 1
MCP-1: Monocyte chemotactic protein-1
CCL-2: Chemokine (C-C motif) ligand-2
PGE2: Prostaglandin E2
TGF-β: Transforming growth factor
S1P: Sphingosine 1-phosphate
LPA: Lysophosphatidic acid
ORG1: Ovarian cancer G-protein-coupled receptor 1
GPR4: G-protein-coupled receptor 4
ERK: Extracellular signal-regulated kinase
LPC: Lysophosphatidylcholine
