# Supplementary material for: A novel sphingosylphosphorylcholine and sphingosine-1-phosphate receptor 1 antagonist, KRO-105714, for alleviating atopic dermatitis
Source: J Inflamm (Lond). 2020 May 29;17:20. doi: 10.1186/s12950-020-00244-6 (PMC7257206; doi:10.1186/s12950-020-00244-6)
Supplement: Supplementary file 1 — Additional file 1: Supplementary data. A novel sphingosylphosphorylcholine and sphingosine-1-phosphate receptor 1 antagonist, KRO-105714, for alleviating atopic dermatitis. Supplementary figure 1. NMR results of KRO-105714 compound. Supplementary figure 2. LC-MS/HRMS result of KRO-105714 compound. Supplementary figure 3. Liver toxicity and alanine aminotransferase activity (ALT) of KRO-105714. Supplementary table 1. Hematotoxicity and hERG potassium channel binding assay of KRO-105714. [file 12950_2020_244_MOESM1_ESM.zip › SPC_supplymentary data_2020.04.16.pdf]

Supplementary Information

A novel sphingosylphosphorylcholine and sphingosine-1-phosphate receptor 1 antagonist, KRO-105714, for alleviating atopic dermatitis

Sae-Bom Yoon, Chang Hoon Lee, Hyun Young Kim, Daeyoung Jeong, Moon Kook Jeon, Sun-A Cho, Kwangmi Kim, Taeho Lee, Jung Yoon Yang, Young-Dae Gong, Heeyeong Cho

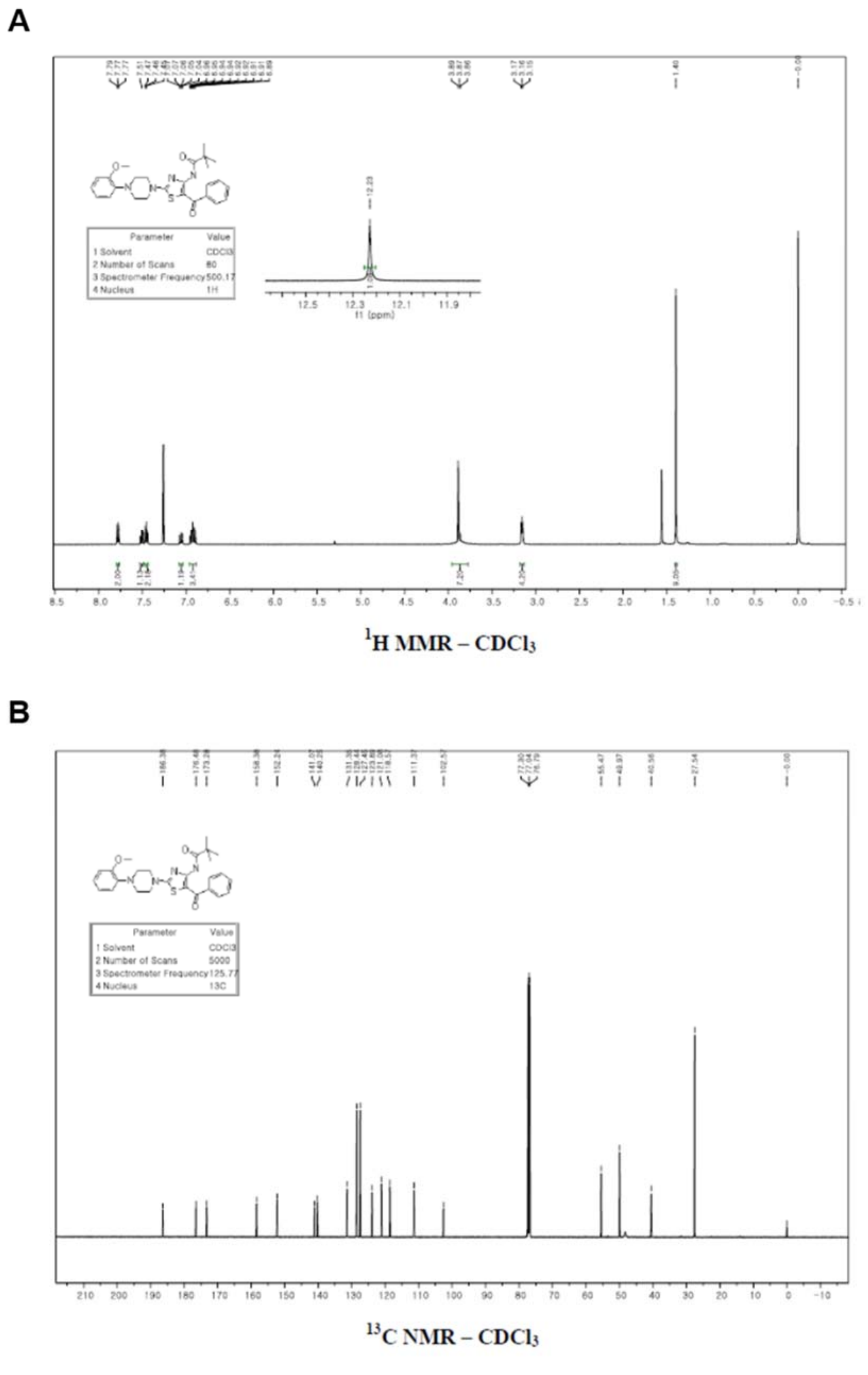

Supplementary figure 1. NMR results of KRO-105714 compound. (A)^{1}H NMR (500 MHz,

$CDCl_3$) δ 12.23 (s, 1H), 7.80 – 7.76 (m, 2H), 7.53 – 7.49 (m, 1H), 7.48 – 7.43 (m, 2H), 7.08 – 7.04 (m, 1H), 6.96 – 6.88 (m, 3H), 3.86-3.89 (m, 7H), 3.18 – 3.13 (m, 4H), 1.40 (s, 9H). (B)^{13}C NMR (126 MHz, $CDCl_3$) δ 186.38, 176.48, 173.28, 158.38, 152.24, 141.07, 140.25, 131.35, 128.44, 127.45, 123.89, 121.08, 118.57, 111.37, 102.57, 77.30, 77.04, 76.79, 55.47, 49.97, 40.56, 27.54, -0.00.

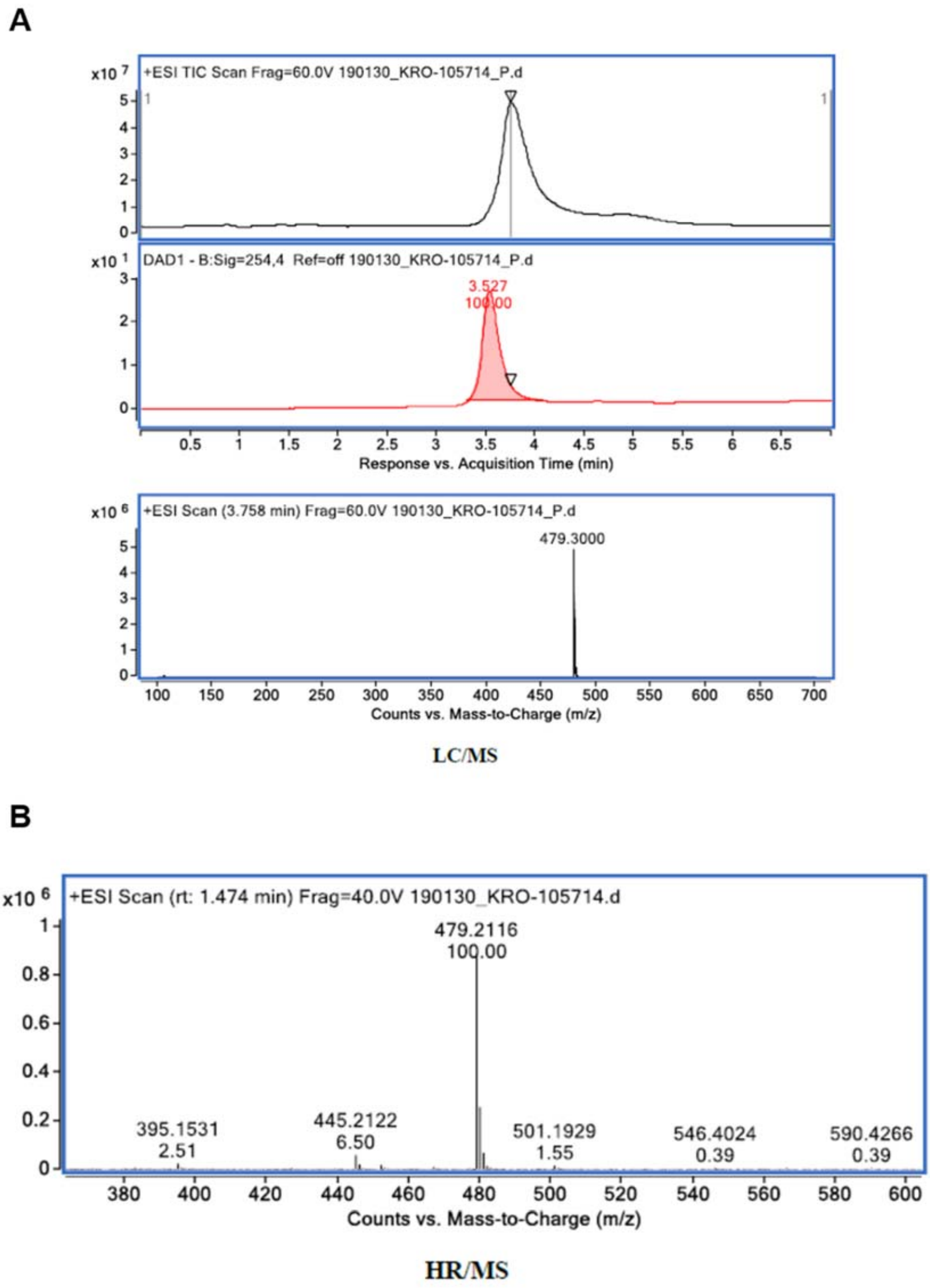

Supplementary figure 2. LC-MS/HRMS result of KRO-105714 compound. LC-MS (ESI): m/z = 479.300 $[M + H]^+$: HRMS (ESI) calculated for $C_{26}H_{30}N_4O_3S$:478.2039, found $[M + H]^+$:

479.21116.

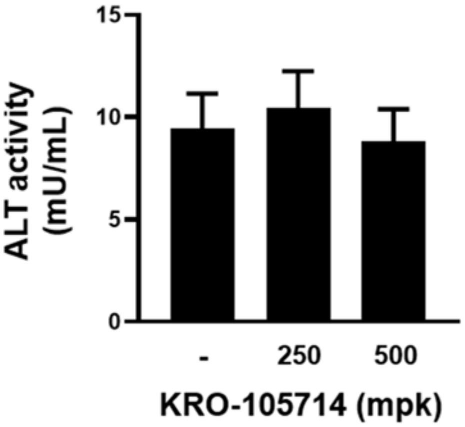

Supplementary figure 3. Liver toxicity and alanine aminotransferase activity (ALT) of KRO-105714. KRO-105714 did not show any changed ALT activity in 250 uM and 500 uM KRO-105714 oral administration.

A

sample ID	IC_{50} (μM)				
	**1A2**	**2C9**	**2C19**	**2D6**	**3A4**
**KRO-105714**	> 10	> 10	> 10	> 10	> 10
**reference**	**α-naphtoflavone**	**sulfaphenazole**	**miconazole**	**quinidine**	**ketoconazole**
	0.20	0.035	< 0.0001	0.0018	0.19
+ ctrl (avg)	0% inhibition 기준 (0.1% DMSO or 0.2% DMSO)				
- ctrl (avg)	100% inhibition 기준 (0.1% or 0.2% DMSO with ctrl membrane)				

B

sample ID		%-Inhibition/[3H]Astemizole				IC_{50} (μM)
		-5	-6	-7	-8	
**KRO-105714**		45	3	< 0	< 0	> 10
**reference**	**Terfenadine**					0.56

Supplementary table 1. Hematotoxicity and hERG potassium channel binding assay of KRO-105714. KRO-105714 showed low hematotoxicity with 1A2: >10 mM, 2C9: >10 mM, 2C19: >10 mM, 2D6: >10 mM, 3A4: >10 mM (IC_{50}) and had no cardiotoxicity in the hERG potassium channel binding assay (IC_{50} >10 mM).
