# Supplementary material for: A novel sphingosylphosphorylcholine and sphingosine-1-phosphate receptor 1 antagonist, KRO-105714, for alleviating atopic dermatitis
Source: J Inflamm (Lond). 2020 May 29;17:20. doi: 10.1186/s12950-020-00244-6 (PMC7257206; doi:10.1186/s12950-020-00244-6)
Supplement: Supplementary file 1 — Additional file 1: Supplementary data. A novel sphingosylphosphorylcholine and sphingosine-1-phosphate receptor 1 antagonist, KRO-105714, for alleviating atopic dermatitis. Supplementary figure 1. NMR results of KRO-105714 compound. Supplementary figure 2. LC-MS/HRMS result of KRO-105714 compound. Supplementary figure 3. Liver toxicity and alanine aminotransferase activity (ALT) of KRO-105714. Supplementary table 1. Hematotoxicity and hERG potassium channel binding assay of KRO-105714. [file 12950_2020_244_MOESM1_ESM.zip › SPC_supporting document.pdf]

Supplementary information

A novel sphingosylphosphorylcholine and sphingosine-1-phosphate receptor 1 antagonist, KRO-105714, for alleviating atopic dermatitis

Sae-Bom Yoon, Chang Hoon Lee, Hyun Young Kim, Daeyoung Jeong, Moon Kook Jeon, Sun-A Cho, Kwangmi Kim, Taeho Lee, Jung Yoon Yang, Young-Dae Gong, Heeyeong Cho

A

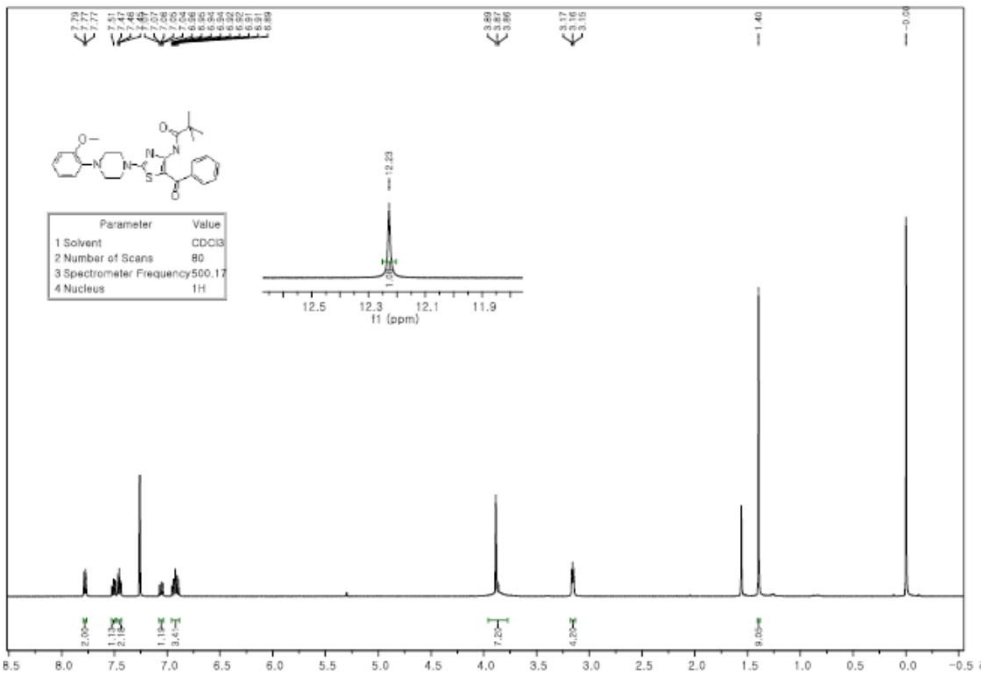

^{1}H MMR – CDCl$_3$

B

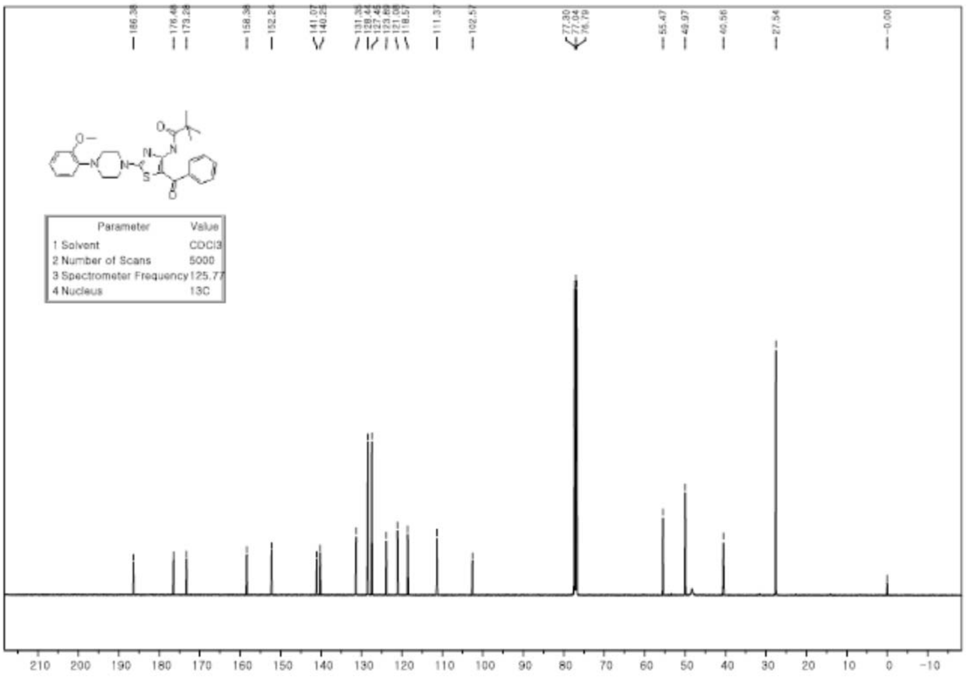

^{13}C NMR – CDCl$_3$

Supplementary figure 1. NMR result of KRO-105714 compound. (A)^{1}H NMR (500 MHz, CDCl$_3$) δ 12.23 (s, 1H), 7.80 – 7.76 (m, 2H), 7.53 – 7.49 (m, 1H), 7.48 – 7.43 (m, 2H), 7.08 – 7.04 (m, 1H), 6.96 – 6.88 (m, 3H), 3.86-3.89 (m, 7H), 3.18 – 3.13 (m, 4H), 1.40 (s, 9H) (Figure S1A). (B)^{13}C NMR (126 MHz, CDCl$_3$) δ 186.38, 176.48, 173.28, 158.38, 152.24, 141.07, 140.25, 131.35, 128.44, 127.45, 123.89, 121.08, 118.57, 111.37, 102.57, 77.30, 77.04, 76.79, 55.47, 49.97, 40.56, 27.54, -0.00.

A

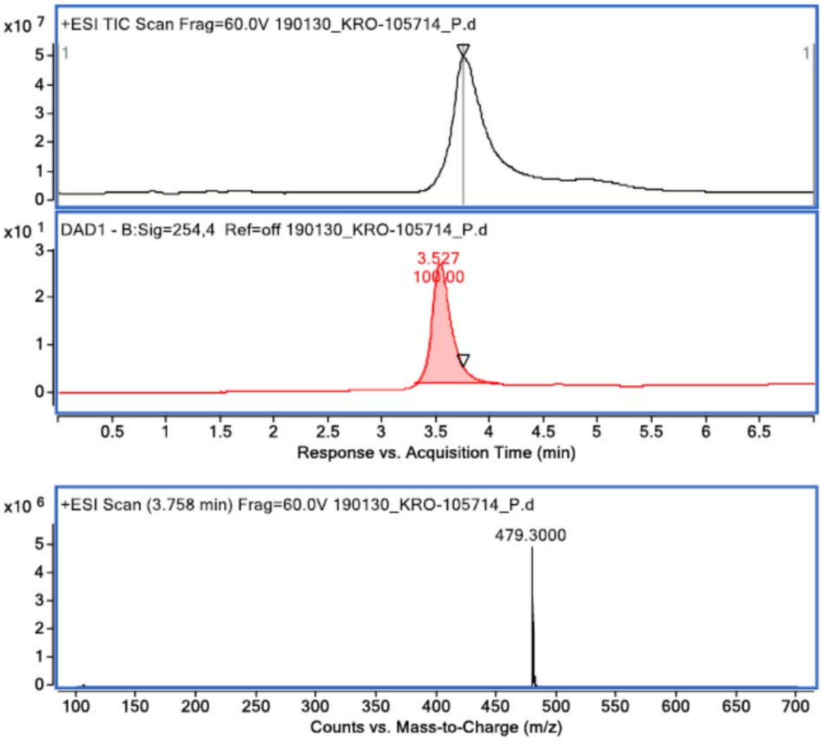

LC/MS

B

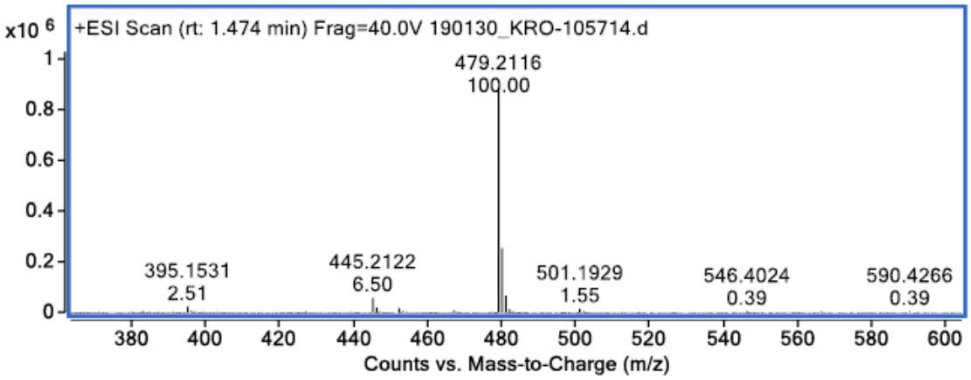

HR/MS

Supplementary figure 2. LC-MS/ HRMS result of KRO-105714 compound.
LC-MS (ESI): m/z = 479.300 $[M + H]^+$: HRMS (ESI) calculated for $C_{26}H_{30}N_4O_3S$: 478.2039, found $[M + H]^+$: 479.21116

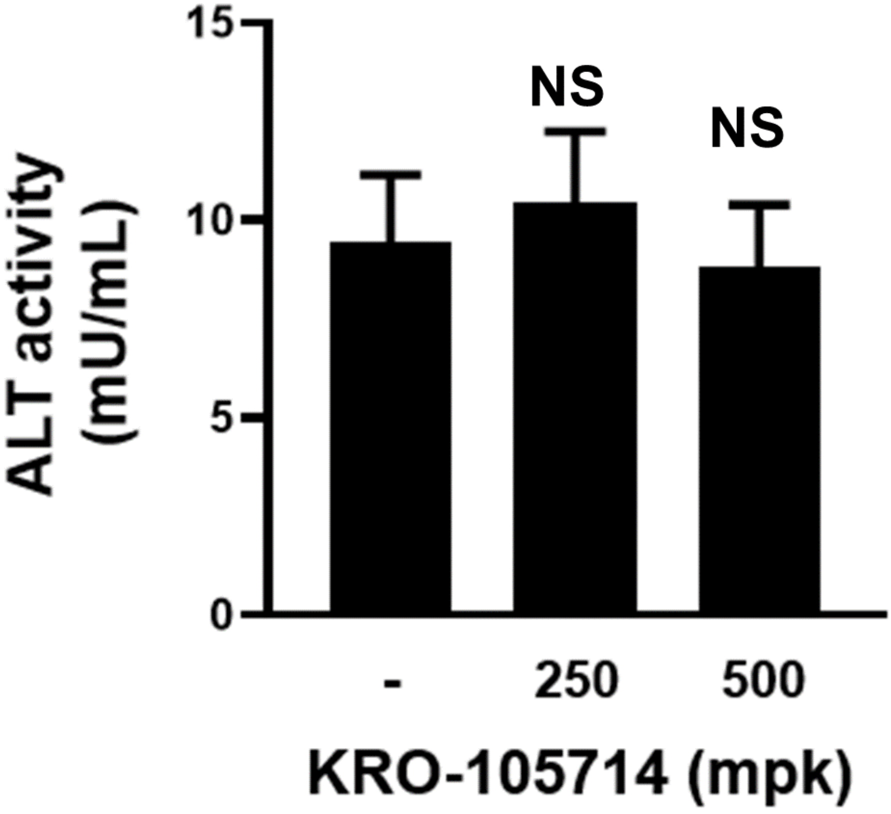

Supplementary figure 3. Liver toxicity and alanine aminotransferase activity (ALT) of KRO-105714. KRO=105714 did not show any changed ALT activy in 250 uM and 500 uM KRO-105713 oral administration.

[illegible]	1A2	2C9	2C19	2D6	3A4
**KRO-105714**	> 10	> 10	> 10	> 10	> 10
**reference**	**α-naphtoflavone**	**sulfaphenazole**	**miconazole**	**quinidine**	**ketoconazole**
	0.20	0.035	< 0.0001	0.0018	0.19
+ ctrl (avg)	0% inhibition 기준 (0.1% DMSO or 0.2% DMSO)				
- ctrl (avg)	100% inhibition 기준 (0.1% or 0.2% DMSO with ctrl membrane)				

B

**sample ID**		**%-Inhibition/[3H]Astemizole**				**IC_{50} (μM)**
		-5	-6	-7	-8	
**KRO-105714**		45	3	< 0	< 0	> 10
**reference**	**Terfenadine**					0.56

Supplementary table 1. Hematotoxicity and hERG potassium channel binding assay of KRO-105714. KRO=105714 showed low hematotoxicity with 1A2: >10 mM, 2C9: >10 mM, 2C19: >10 mM, 2D6: >10 mM, 3A4: >10 mM (IC_{50}) and had no cardiotoxicity in the hERG potassium channel binding assay (IC_{50} >10 mM)
